# Identification of an immune-related signature indicating the dedifferentiation of thyroid cells

**DOI:** 10.1186/s12935-021-01939-3

**Published:** 2021-04-23

**Authors:** Xuemin Wang, Wen Peng, Chunyan Li, Rujia Qin, Zhaoming Zhong, Chuanzheng Sun

**Affiliations:** 1 Department of Head and Neck Surgery Section II, The Third Affiliated Hospital of Kunming Medical University/Yunnan Cancer Hospital, 519 Kunzhou Road, Kunming, China; 2 Department of Medical Oncology, The First Affiliated Hospital of Kunming Medical University, 295 Xichang Road, Kunming, China

**Keywords:** Dedifferentiation, Anaplastic thyroid carcinoma, Papillary thyroid carcinoma, Immune-related genes, Prognosis, Infiltrating immune cells, Immune checkpoints, Immunotherapy

## Abstract

**Background:**

Immune cells account for a large proportion of the tumour microenvironment in anaplastic thyroid carcinomas (ATCs). However, the expression pattern of immune-related genes (IRGs) in ATCs is unclear. Our study aimed to identify an immune-related signature indicating the dedifferentiation of thyroid cells.

**Methods:**

We compared the differences in thyroid differentiation score (TDS), infiltration of immune cells and enriched pathways between ATCs and papillary thyroid carcinomas (PTCs) or normal thyroid tissues in the Gene Expression Omnibus database. Univariate and multivariable Cox analyses were used to screen prognosis-associated IRGs in The Cancer Genome Atlas database. After constructing a risk score, we investigated its predictive value for differentiation and survival by applying receiver operating characteristic and Kaplan–Meier curves. We further explored its associations with important immune checkpoint molecules, infiltrating immune cells and response to immunotherapy.

**Results:**

Compared with PTCs or normal thyroid tissues, ATCs exhibited lower TDS values and higher enrichment of immune cells and activation of the inflammatory response. The quantitative analyses and immunohistochemical staining validated that most ATC cell lines and ATC tissues had higher expression of MMP9 and lower expression of SDC2 than normal thyroid samples and PTC. Higher risk scores indicates dedifferentiation and a worse prognosis. Additionally, the risk score was positively correlated with the immune checkpoint molecules PDL1, CTLA4, IDO1, and HAVCR2 and infiltration of multiple immune cells. Importantly, we found that the samples with higher risk scores tended to have a better response to immunotherapy than those with lower scores.

**Conclusion:**

Our findings indicate that the risk score may not only contribute to the determination of differentiation and prognosis of thyroid carcinomas but also help the prediction of immune cells infiltration and immunotherapy response.

**Supplementary Information:**

The online version contains supplementary material available at 10.1186/s12935-021-01939-3.

## Background

Thyroid carcinoma is a common endocrine malignancy [1]. Among all subtypes, papillary thyroid carcinoma (PTC) accounts for approximately 90%, and most patients can achieve long-term survival after reasonable treatments [2]. In contrast, anaplastic thyroid carcinoma (ATC) is an undifferentiated tumour that is extremely rare and highly aggressive, with a dismal median survival of only 4 months [3]. Conventional systemic treatments, including radioiodine therapy, radiotherapy and chemotherapy, have a limited effect on ATC, rendering ATC a significant clinical challenge [4]. An important hallmark of ATC is the deficient expression of thyroid differentiation markers, which partially leads to radioiodine therapy failure [5].

The most accepted hypothesis claims that ATC progresses from PTC through the accumulation of many genomic mutations [5, 6]. However, Seoane et al. [7] performed exome sequencing and found very few shared trunk alterations between ATC and PTC, leading these authors to believe that ATC and PTC evolve independently at an early stage of tumour development. This finding implies that the clinical management of the two tumours should be appropriate for their molecular characteristics, requiring a deep understanding of the undifferentiated feature of ATC.

Recently, extensive studies have revealed the essential role of the tumour microenvironment (TME) in cancer progression and treatment [8, 9]. ATC has been reported to be greatly infiltrated by tumour-associated macrophages (TAMs) and possesses high levels of M2 macrophage phenotype markers, which promote tumour metastasis [5, 10–12]. Additionally, some immune checkpoint inhibitors have shown effects on ATC [13–16], prompting us to investigate the role of immune cells or immune-related genes (IRGs) in the occurrence and progression of ATC.

In our study, by exploring the expression patterns of IRGs in ATCs, PTCs and normal thyroid tissues in the Gene Expression Omnibus (GEO) and The Cancer Genome Atlas (TCGA) databases, a two-gene risk score signature comprising MMP9 and SDC2 with predictive power for dedifferentiation and survival was constructed. Additionally, significant correlations were found between the risk score and important immune checkpoints or infiltrating immune cells. Moreover, the risk score was found to have a certain predictive value for the immunotherapy response.

## Materials and methods

### Public cohort datasets and preprocessing

We systematically searched for publicly available ATC transcriptome datasets. In total, the following four cohorts using the same array platform (Affymetrix Human Genome U133 Plus 2.0 Array) were gathered for this study: GSE33630, GSE29265, GSE65144 and GSE76039 [5, 17, 18]. The raw expression data were downloaded from the GEO database (https://www.ncbi.nlm.nih.gov/geo/). The boxplot function in R package was used to determine whether the distribution of the samples’ expression abundance value is uniform. If not, the normalizeBetweenArrays function in the R package limma was applied to correct the expression data, which were then log2 transformed. When a gene symbol corresponded to multiple probes, we selected the highest value as its expression level, and a probe was deleted when it was recorded with multiple genes. To enhance the reliability of the validation of the screened genes, we merged the GEO datasets mentioned above, and batch effects were removed using the R package sva.

We also used transcriptome data and clinical information of PTCs from the TCGA database downloaded from the UCSC Xena browser (https://xenabrowser.net/datapages/), and 505 patients with PTC were enrolled in our study. Considering the excellent survival outcome in most PTC patients, the progression-free interval (PFI) was regarded as the preferable indicator of prognosis [19].

To investigate the treatment response to immunotherapy, a dataset of urothelial cancer patients who received anti-PD-L1 therapy (IMvigor210 cohort) was acquired from the R package IMvigor210CoreBiologies, and a dataset of AB1-HA mesothelioma mice treated with anti-CTLA4 agents (GSE63557) was obtained [20, 21].

To compare the differentiation level among the different samples, a list of 16 TDS genes was obtained from a published study investigating PTC and served as a parameter of thyroid differentiation [22]. We summed the 16 genes in each sample to obtain the TDS and then separated the PTCs from the TCGA dataset into a low-differentiated group and a high-differentiated group according to the median value.

### Collection of immune related data

The stromal score and immune score of each sample were calculated by the ESTIMATE package in the R program [23]. Single sample gene set enrichment analysis(ssGSEA), as implemented in the R package GSVA, was used to assess the enrichment levels of 29 immune cells, and a principal component analysis (PCA) was applied.

The lists of IRGs were collected from the ImmPort web portal (https://immport.org/shared/home), which contains vast immunology data and resources. Overall, 1240 IRGs were present in our array and were further analysed.

### Function and pathway enrichment analysis

A gene annotation enrichment analysis was carried out by using the R package clusterProfiler. A gene set enrichment analysis (GSEA) was performed three times in our study as follows: one GSEA was performed to compare the differences in IRGs between ATCs and normal tissues or PTCs in the GSE33630 cohort; the second GSEA was performed to identify differentiation-associated immune signalling pathways in the TCGA cohort; and the third was to analyse the differences between the low-risk-score and high-risk-score subtypes in the expression of broad hallmark gene sets in the combined GEO cohort [24]. We also identified the signalling pathways of the deregulated IRGs in ATCs in the GSE33630 cohort by performing Gene Ontology (GO) and Kyoto Encyclopedia of Genes and Genomes (KEGG) enrichment analyses.

### Cell culture

All cell lines were cultured in DMEM supplemented with 10% fetal bovine serum (FBS) (Gibco). The normal thyroid follicular epithelial cell line Nthy-ori 3-1 and PTC cell line TPC-1 were obtained from the Institute of Medical Biology Chinese Academy of Medical Sciences (Kunming). The ATC cell lines CAL-62, DRO and 8305C were provided by Sun Yat-sen University Cancer Center (Guangzhou). All cell lines were authenticated by short tandem repeat (STR) sequencing and confirmed to be negative of mycoplasma.

### qRT-PCR

The total RNA was isolated from the cells using a FastPure® Cell/Tissue Total RNA Isolation Kit (Vazyme). cDNA synthesis was carried out using HiScript® II Q RT SuperMix (Vazyme). qPCR was performed using ChamQ™ SYBR® qPCR Master Mix (Vazyme) on an Applied Biosystems 7500 system. The reaction procedure was performed according to the manufacturer’s instructions. The sequences of the primers were as follows:

- human GAPDH forward: CTCCTGCACCACCAACTGCT,
- human GAPDH reverse: GGGCCATCCACAGTCTTCTG;
- human MMP9 forward: CAGTCCACCCTTGTGCTCTTC,
- human MMP9 reverse: TGCCACCCGAGTGTAACCAT;
- human SDC2 forward: TGGAAACCACGACGCTGAATA,
- human SDC2 reverse: ATAACTCCACCAGCAATGACAG.

GAPDH was used as a control, and the fold changes of the target genes were calculated by the ΔΔCt method; the normalized gene expression was calculated using 2^−ΔΔCt^.

### Immunohistochemical staining

Paraffin-embedded specimens of 11 ATCs, 20 PTCs and 20 normal thyroid tissues were sectioned at 4 µm and incubated at 70 °C for 2 h. Xylene was used to deparaffinize the samples, and then, the sections were hydrated in gradient ethanol. Antigen retrieval was carried out using sodium citrate and a pressure cooker for boiling for 2 min. After washing with phosphate-buffered saline (PBS), the slides were incubated with the following primary antibodies overnight: anti-SDC2 (Proteintech, 1:300), anti-MMP9 (Cell Signaling Technology, 1:100), anti-TG (Proteintech, 1:200), anti-PLAUR (Proteintech, 1:100) and anti-FGFR2 (Cell Signaling Technology, 1:100). After incubating with the appropriate secondary antibodies, the samples were reacted with DAB reagents. Immunohistochemistry (IHC) score included the staining intensity and the percentage of positive staining. The expression intensity was scored as follows: 0 (no), 1 (weak), 2 (moderate), 3 (strong). The percentage was scored as follows: 0 (0–5%), 1 (6–25%), 2 (26–50%), 3 (51–75%), 4 (75–100%). The final IHC score was calculated by multiplying the intensity score by the proportion score. The details of the IHC score for each protein are provided in Additional file 5: Table S2.

### Statistical analysis

The analysis of the count data of differentially expressed IRGs was performed by the R package limma in the GEO cohorts and IMvigor210 cohort and the R package DESeq2 in TCGA cohort. The Benjamini–Hochberg method was applied to adjust the p-value based on the false discovery rate (FDR). The eBayes method in the R package was used to identify the differentially expressed genes (DEGs), with an FDR < 0.05 and fold change ≥ 2 as screening conditions. The R packages VennDiagram and Heatmap were used to draw Venn diagrams and heatmaps. ggplot2 was used to generate volcano plots and other plots.

The Wilcoxon test (also called the Mann–Whitney U test) was applied to compare the continuous variables between the two groups. For comparisons among three groups, the Kruskal–Wallis test was used. To evaluate the association between the clinicopathological characteristics or IRGs and PFI in the TCGA cohort, a univariate Cox proportional hazard model was used. A multivariable Cox regression model was further conducted to identify the independent prognostic factors. Both the hazard ratio (HR) and 95% confidence interval (CI) were calculated.

To investigate the role of selected IRGs, a risk score model was constructed by integrating the regression coefficient derived from the multivariable Cox regression analysis and the expression data of screened IRGs as follows: risk score = ∑(Coefi * Expi) [25]. Based on the median value, the samples from different cohorts were dichotomized into a low-risk score group and a high-risk score group. Correlation analyses were conducting by using Pearson’s test in the R program. Receiver operating characteristic (ROC) analyses were carried out to calculate the area under the curve (AUC) to assess the accuracy of the risk score in predicting dedifferentiation. Kaplan–Meier curves of the PFI were built, and the log-rank test was applied to determine the differences using SPSS software. The distributions of the response to immunotherapy in the low-risk score group and high-risk score group were evaluated with a chi-square test or two-sided Fisher’s exact test.

All graphic representations and statistical analyses were performed by using R software (version 3.6.1), GraphPad Prism (version 7.0) and SPSS software (version 21.0). p < 0.05 was considered statistically significant in all analyses.

## Results

### ATCs exhibit lower TDS values and higher infiltration levels of most immune cells than PTCs and normal thyroid tissues

**Fig. 1:**
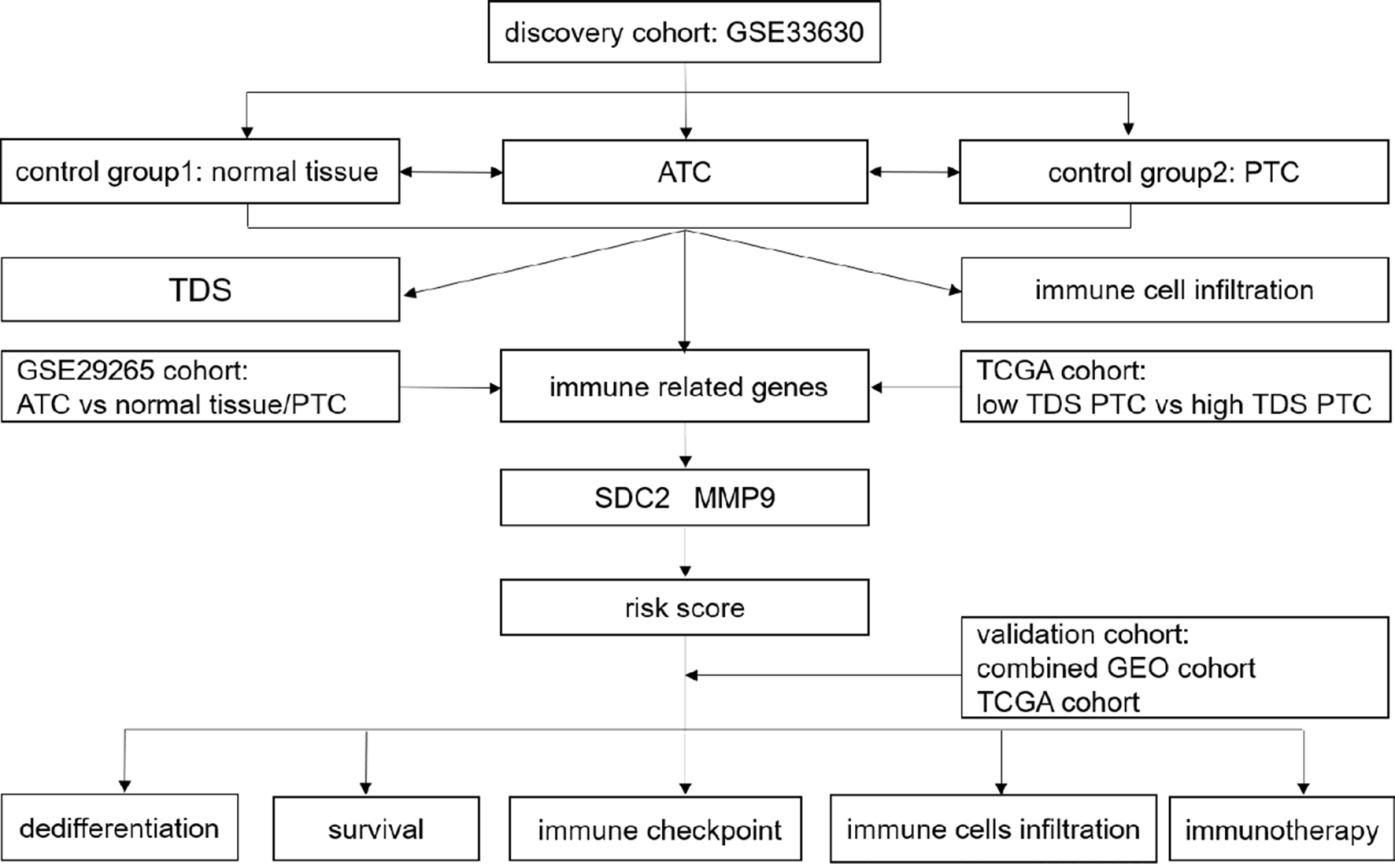
Flow chart of the analysis strategy of our study

**Fig. 2:**
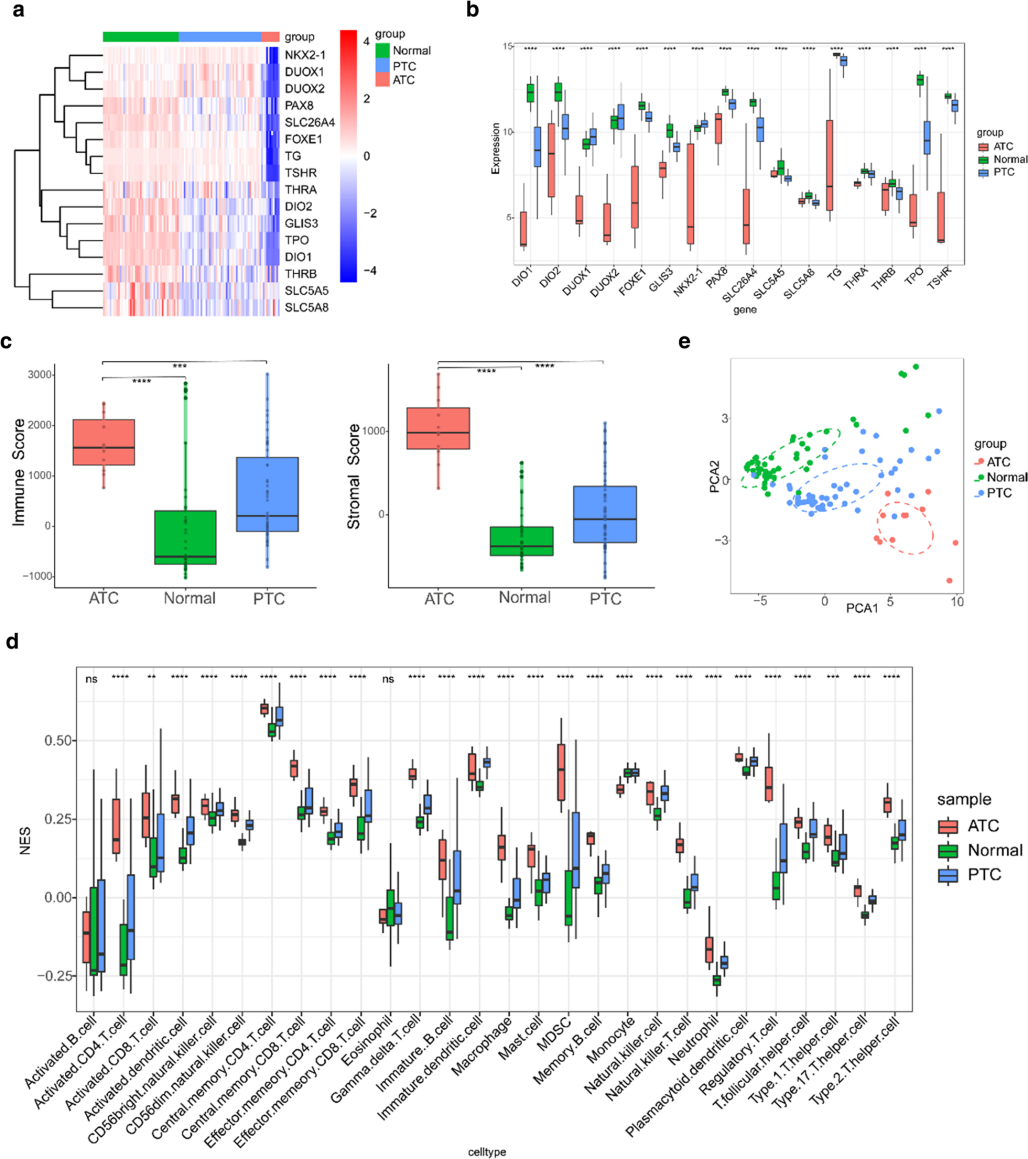
Comparison of TDS and immune cell infiltration among ATCs, PTCs and normal tissues. **a**, **b** Based on a 16-gene TDS signature, ATCs had obviously lower TDS values than PTCs or normal thyroid tissues in all genes as shown in the heatmap (**a**) and boxplot (**b**) (Kruskal–Wallis test). **c** Comparison of immune scores and stromal scores between ATCs and PTCs or normal tissues (Wilcoxon test). **d** The infiltration level of most immune cells in ATCs was higher than that in PTCs and normal tissues. **e** PCA discriminated ATCs from PTCs and normal tissues (Kruskal–Wallis test). *ns* not significant; *p-value < 0.05; **p-value < 0.01; ***p-value < 0.001; ****p-value < 0.0001

An overview of our study is shown in Fig. 1. We initially compared the TDS levels among ATCs, PTCs and normal thyroid tissues in the GSE33630 cohort. As expected, ATCs had obviously lower TDS values than the control samples of all genes (Fig. 2a, b). It has been reported that the immune landscape is correlated with TDS in PTCs [26]. The ESTIMATE algorithm and GSVA package in R program were applied to evaluate the differences in immune-related signatures and showed that ATCs had higher stromal scores, immune scores and enrichment of most immune cells than the control groups (Fig. 2c, d). Moreover, ATCs were well discriminated from PTCs or normal tissues with the PCA algorithm (Fig. 2e).

### The inflammatory response is obviously activated in ATCs

**Fig. 3:**
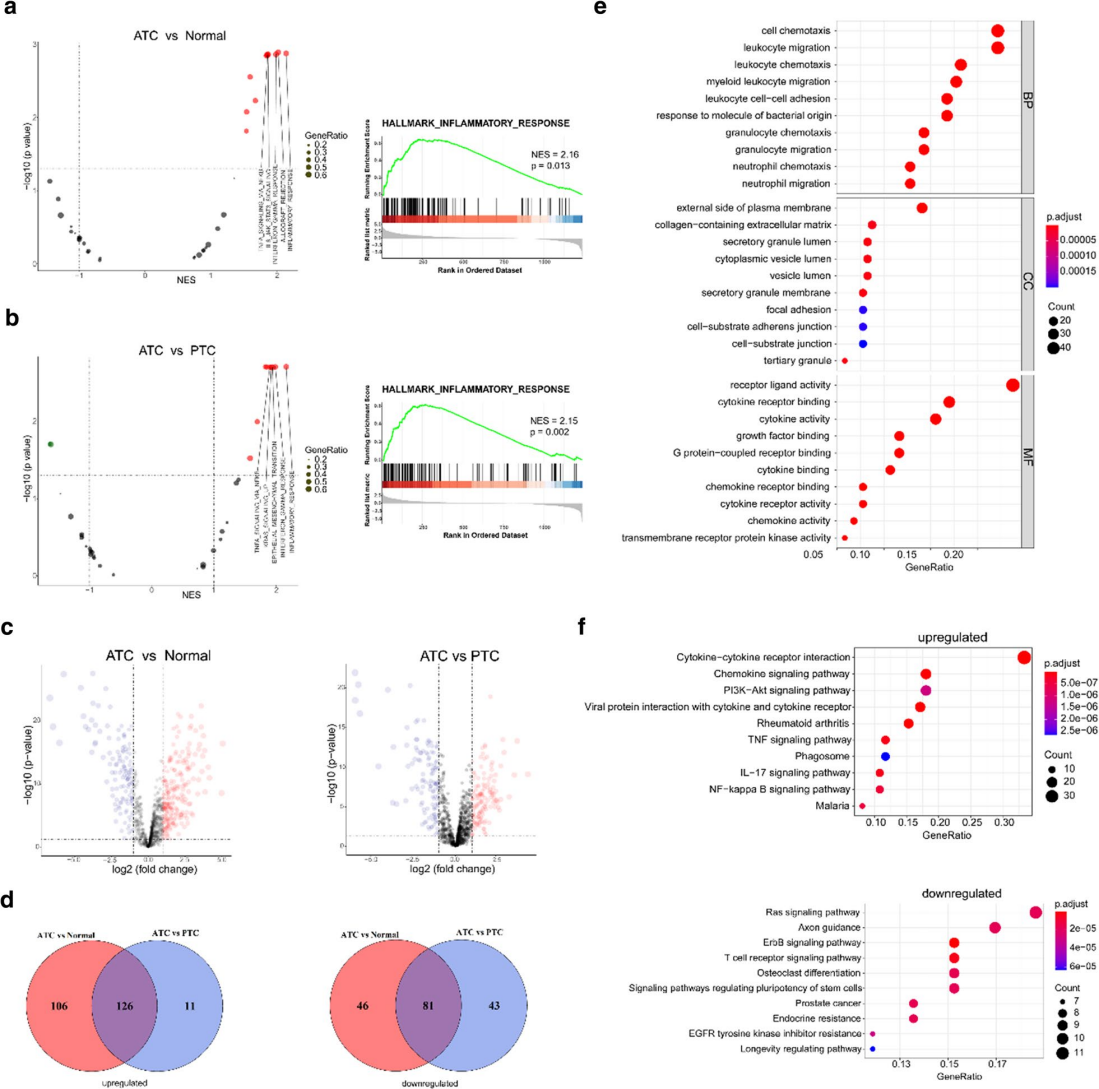
Exploration of the function of IRGs in ATCs, PTCs and normal tissues. **a**, **b** GSEA of IRGs showed the top 5 activated pathways (left panels) in ATCs compared with normal tissues (**a**) and PTCs (**b**); the inflammatory response (right panels) was the most enriched. **c** Volcano plots of differentially expressed IRGs between ATCs and normal tissues (left panel) or PTCs (right panel). **d** Venn diagram showing the consistently deregulated genes between ATCs and PTCs or normal tissues in the GSE33630 cohort. **e**, **f** GO (**e**) and KEGG (**f**) enrichment analyses of 207 commonly deregulated IRGs

**Fig. 4:**
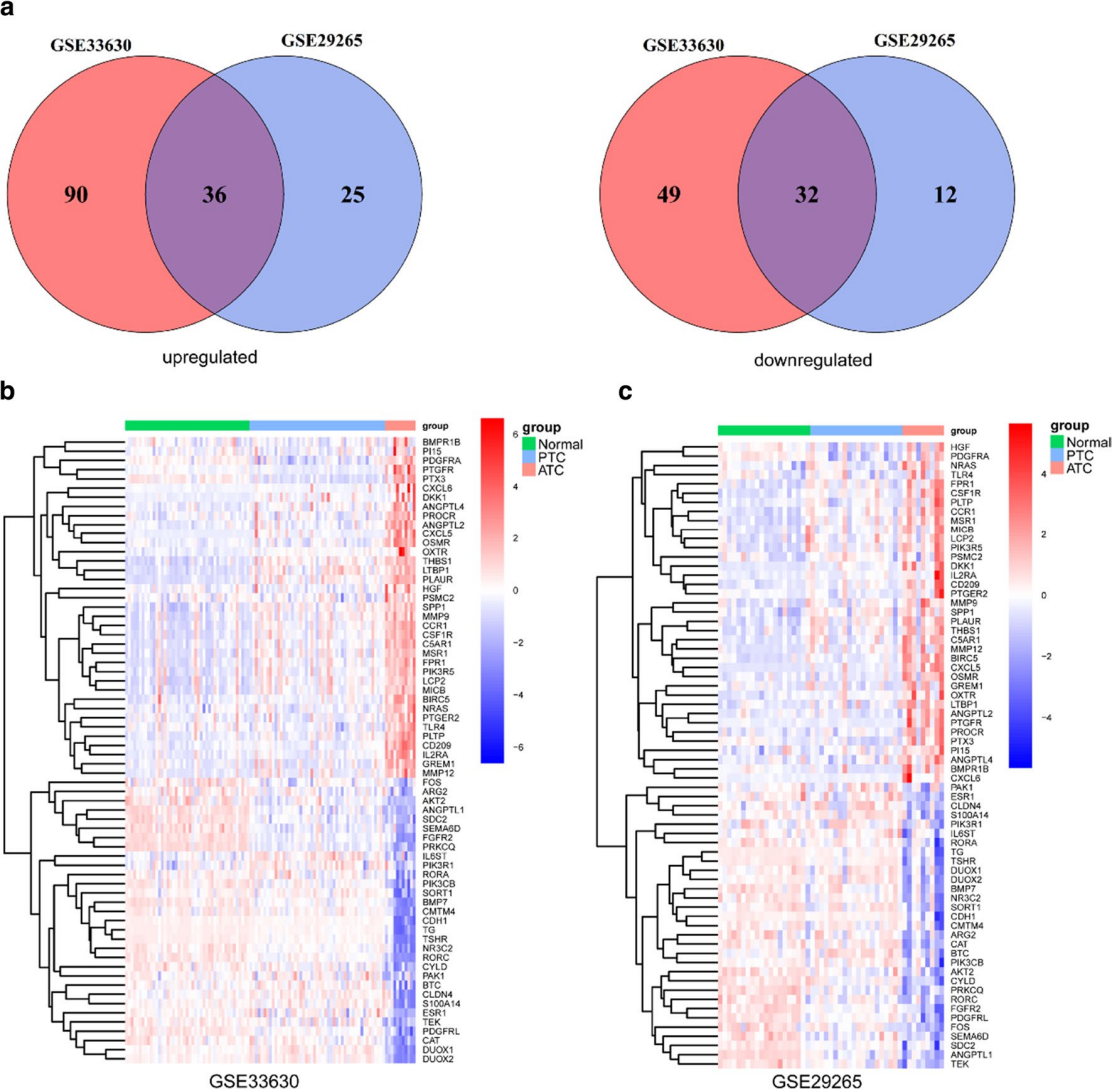
Identification of deregulated IRGs in ATCs compared with PTCs and normal tissues. **a** Overlapping analyses screened 68 deregulated IRGs. **b**, **c** Heatmaps of the expression data of 68 IRGs in the GSE33630 (**b**) and GSE29265 (**c**) cohorts

To further explore the differences in IRGs between ATCs and PTCs or normal thyroid tissues in the GSE33630 cohort, the powerful web tool ImmPort was used, and in total, 1240 IRGs were present in our array. The volcano plots of GSEA displayed the top five pathways significantly activated in ATCs compared with normal tissues or PTCs; among these pathways, the inflammatory response was the most obvious (Fig. 3a, b, Additional file 1: Figure S1). In total, 207 IRGs were found to be commonly deregulated in ATCs compared with both normal tissues and PTCs (Fig. 3c, d). The GO and KEGG enrichment analyses indicated significant activation of immune-associated signalling pathways in ATCs (Fig. 3e, f). To further screen the deregulated IRGs, another GEO cohort, i.e., GSE29265, was included, and in total, 68 genes were screened and validated in the two cohorts (Fig. 4a–c).

### Identification of differentiation-associated and prognostic IRGs

To further analyse the differentiation-associated IRGs, we enrolled 505 PTC samples with complete clinical annotations and transcriptome data from TCGA database, which were then divided into the low-differentiated (low TDS) group and the high-differentiated (high TDS) group by the median TDS value. The volcano plot based on a GSEA of 1240 IRGs indicated that compared with the high-differentiated group, the interferon gamma response (NES = 2.15, p = 0.003) and inflammatory response (NES = 2.10, p = 0.003) were significantly enriched in the low-differentiated group (Additional file 2: Figure S2). Considering that patients with ATC lack clinical information in the GEO database, we focused on the low-differentiated PTCs in TCGA cohort to identify the prognostic IRGs. First, the clinical characteristics and 68 IRGs were included in a univariate Cox analysis. Stage III/IV, T3/T4, distant metastasis and five genes, including matrix metalloproteinase-9 (MMP9), fibroblast growth factor receptor 2 (FGFR2), plasminogen activator, urokinase receptor (PLAUR), syndecan-2 (SDC2) and thyroglobulin (TG), were identified as risk factors (Additional file 3: Table S1). Then, these risk factors were included in a multivariable Cox analysis. Considering the mutual effect among IRGs and clinical characteristics, only SDC2 (HR = 0.614; p = 0.033) and MMP9 (HR = 1.339; p = 0.009) were identified as independent prognostic IRGs of low-differentiated PTCs (Table 1).

**Table 1:** Multivariable analysis showed independent prognosis-associated IRGs for low-differentiated PTCs in TCGA database

Variable	Multivariable analysis			
	Coefficient	HR	95% CI	p
SDC2	− 0.488	0.614	0.391–0.962	0.033
MMP9	0.292	1.339	1.076–1.666	0.009

IRGs were evaluated as continuous variables in multivariable Cox regression analysis

**Fig. 5:**
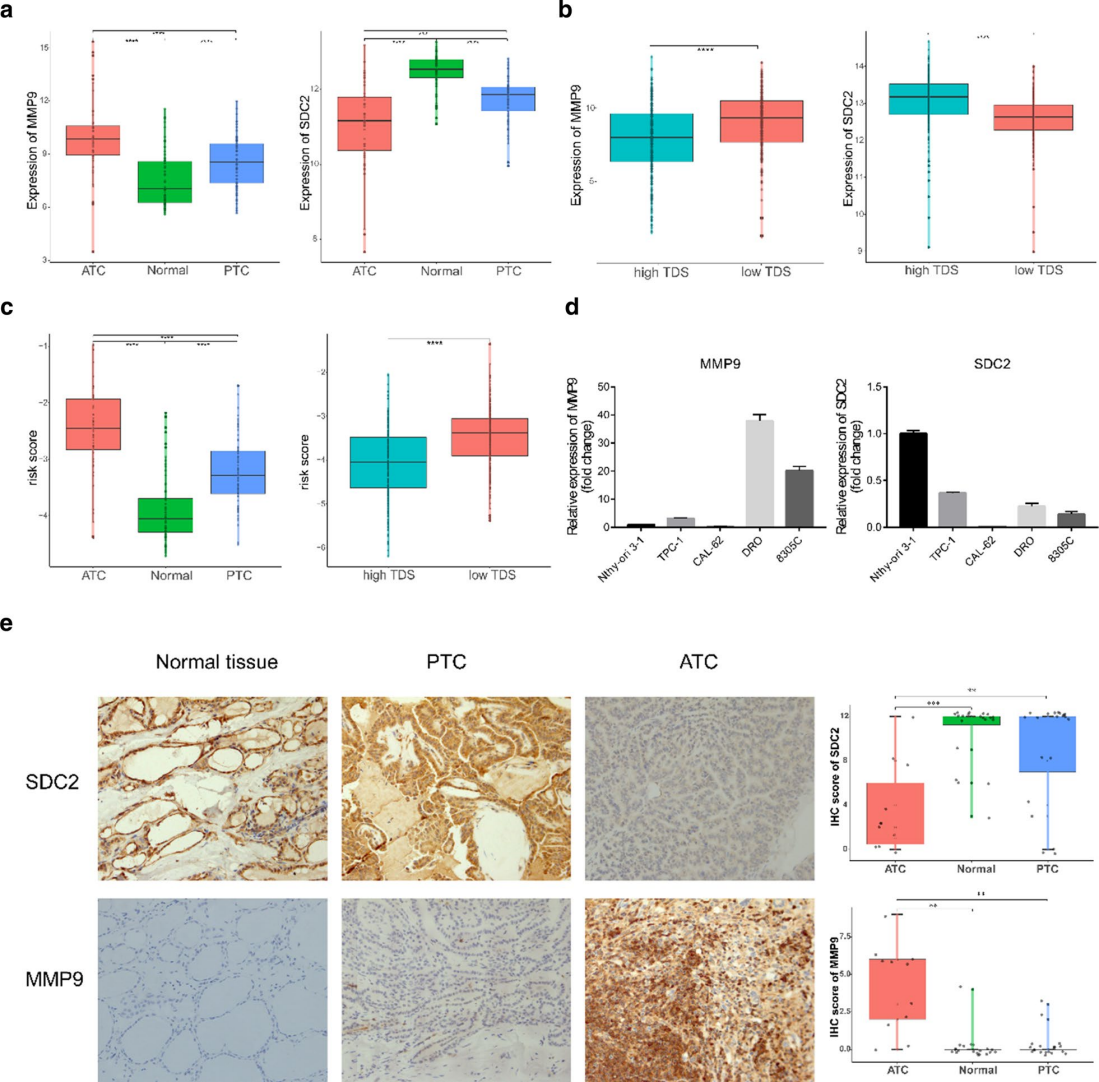
Verification the expression of MMP9 and SDC2. **a**, **b** Expression of MMP9 and SDC2 in the combined GEO cohort (**a**) and TCGA cohort (**b**) (Wilcoxon test). **c** Comparison of risk scores in the combined GEO cohort (left) and TCGA cohort (right) (Wilcoxon test). **d** Relative expression of MMP9 and SDC2 in the Nthy-ori 3-1, TPC-1, CAL-62, DRO, and 8305C cell lines. **e** Representative sections of normal thyroid, PTC and ATC tissues. The expression of SDC2 and MMP9 was detected by using immunohistochemistry (IHC)

Then, we assessed the expression levels of MMP9 and SDC2 in a combined GEO cohort comprising 52 ATCs, 78 normal tissues and 69 PTCs from the same chip platform and TCGA cohort grouped by TDS level. As shown in Fig. 5a, b, the expression of MMP9 in ATCs and low-differentiated PTCs was significantly higher than that in normal tissues and high-differentiated PTCs, whereas SDC2 displayed the opposite expression profile. In addition, an immune-related risk score was constructed and was obviously higher in ATCs and low-differentiated PTCs (Fig. 5c). qPCR analyses further indicated that the ATC cell lines CAL-62, DRO and 8305C possessed lower SDC2 levels; DRO and 8305C had higher MMP9 levels than the PTC cell line TPC-1 and normal thyroid cell line Nthy-ori 3-1 (Fig. 5d). Moreover, the immunohistochemical staining in normal thyroid, PTC and ATC tissues showed higher expression of MMP9 and lower expression of SDC2 in ATC patients (Fig. 5e; Additional file 4: Figure S3; Additional file 5: Table S2).

**Fig. 6:**
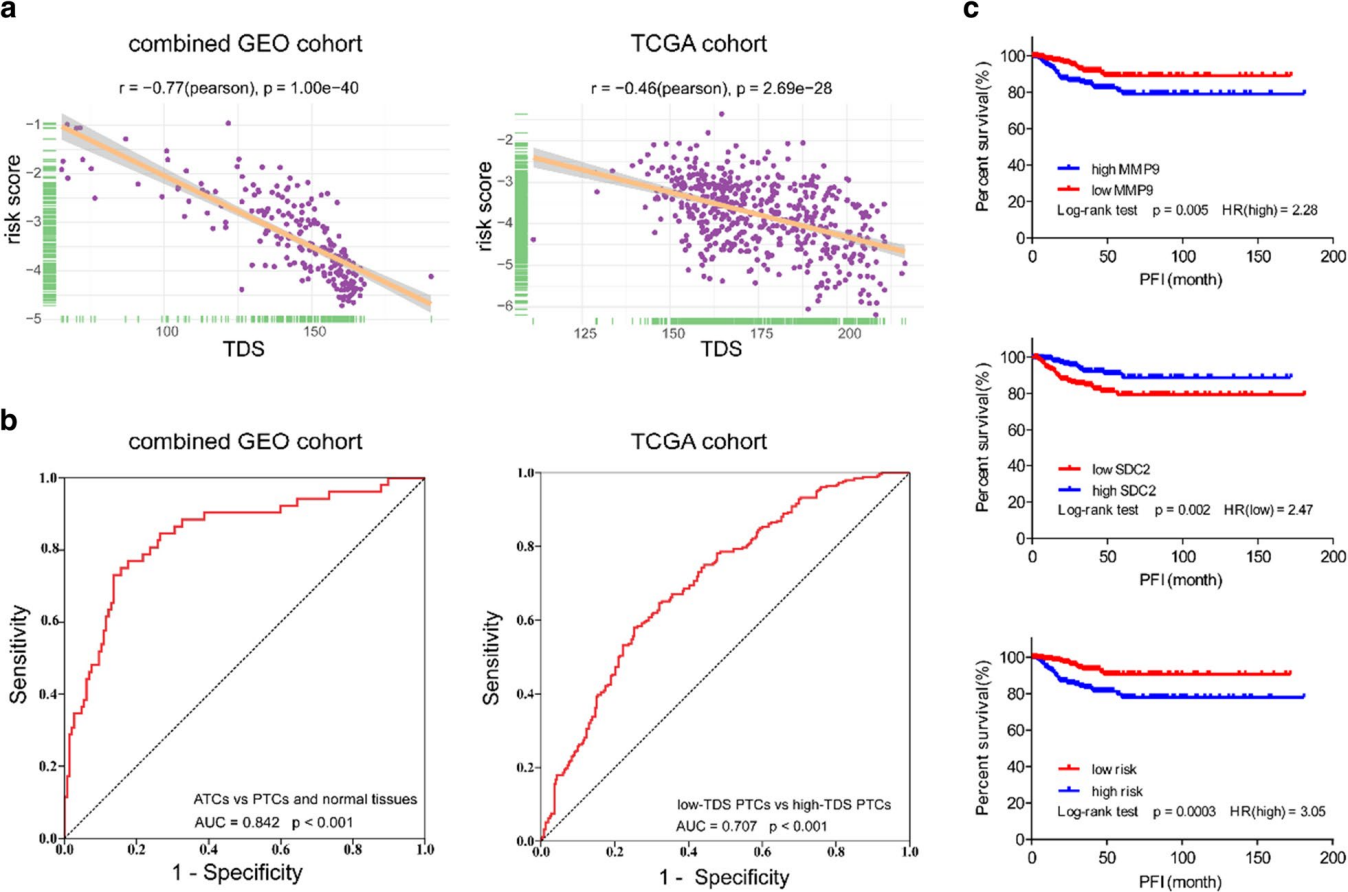
The MMP9 and SDC2 risk score predicts dedifferentiation and a poor prognosis. **a** Scatter plots showing a significant correlation between the risk score and the TDS in the combined GEO cohort and TCGA cohort (Pearson test). **b** ROC curves indicating the power of the risk score in predicting dedifferentiation in the combined GEO cohort and TCGA cohort. **c** Kaplan–Meier curves of the PFI of 505 PTC patients in TCGA cohort grouped by MMP9 expression, SDC2 expression and risk score (log-rank test)

We proceeded to analyse the ability of the risk score to predict dedifferentiation. The correlation analyses showed that the risk score was negatively associated with TDS in both the combined GEO cohort and TCGA cohort (Fig. 6a). Consistent with this finding, the ROC curves confirmed the robust predictive value in both cohorts, and the AUC values were 0.842 (p < 0.001) and 0.707 (p < 0.001) (Fig. 6b). Moreover, we estimated the prognostic value of a single gene and the two-gene risk score. The Kaplan–Meier curves (Fig. 6c) indicated that the PTC patients with high MMP9 (HR = 2.28; p = 0.005), low SDC2 (HR = 2.47; p = 0.002) or a high risk score (HR = 3.05; p = 0.0003) had a significantly shorter PFI than the other patients in the TCGA cohort, indicating the potential of the risk score to serve as a prognostic biomarker.

### The two-gene risk score is closely correlated with immune-related signatures

**Fig. 7:**
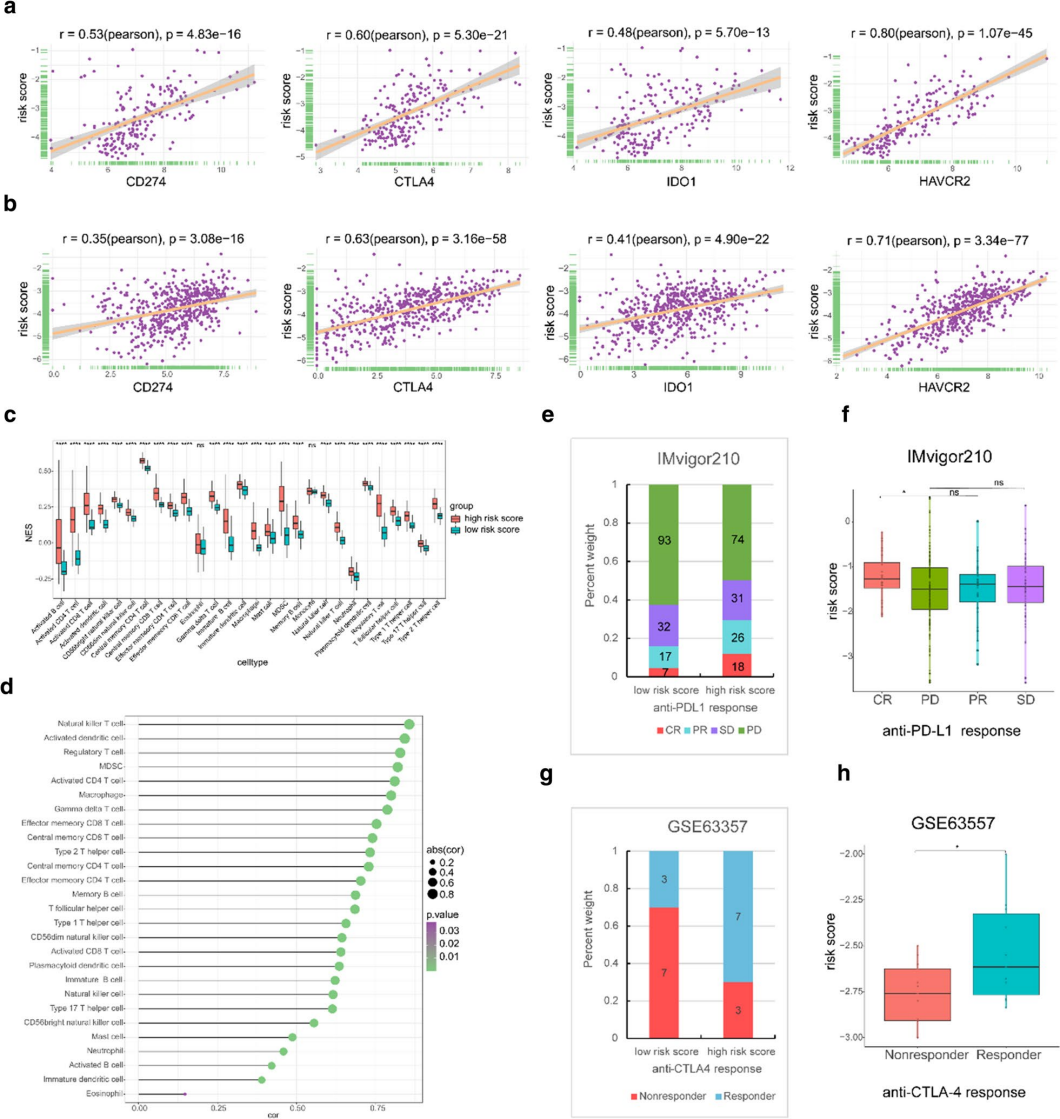
Exploring the role of the risk score in immune-related signatures. **a**, **b** Plots indicating the correlations between the risk score and the immune checkpoint molecules CD274 (PDL1), CTLA4, IDO1, and HAVCR2 (TIM3) in the combined GEO cohort (**a**) and TCGA cohort (**b**) (Pearson test). **c** Comparison of the infiltration of immune cells between the low risk score and high risk score samples in the combined GEO cohort (Wilcoxon test). **d** The association between the risk score and the infiltration of immune cells in the combined GEO cohort (Pearson test). **e** Response to anti-PD-L1 immunotherapy in the low- and high-risk-score groups in the IMvigor210 cohort (chi-square test, p = 0.003) (*CR* complete response, *PR* partial response, *SD* stable disease, *PD* progressive disease). **f** Distribution of the risk score among patients with different anti-PD-L1 treatment responses in the IMvigor210 cohort (Wilcoxon test). **g** Response to anti-CTLA4 immunotherapy in the low- and high-risk-score groups in the GSE63557 cohort (two-sided Fisher’s exact test, p = 0.18). **h** Distribution of the risk score among mice with different anti-CTLA4 treatment responses in the GSE63557 cohort (Wilcoxon test)

Immunotherapy has revolutionized the treatment of many patients with cancer. However, it has been found that some negative costimulatory molecules and immunosuppressive cytokines could promote immune escape in tumour cells by inhibiting immune responses [27, 28], stimulating the appearance of immune checkpoint therapy. We sought to explore the role of the two-gene risk score in immune-related signatures. First, a markedly positive correlation was found between the risk score and important immune checkpoint molecules, including programmed cell death ligand 1 (PDL1; also known as CD274), cytotoxic T-lymphocyte-associated protein 4 (CTLA4), indoleamine 2,3-dioxygenase 1 (IDO1) and hepatitis A virus cellular receptor 2 (HAVCR2; also known as TIM3) in both the combined GEO cohort and TCGA cohort (Fig. 7a, b). Moreover, we found that most immune cells displayed higher enrichment score in the high-risk score group than the low-risk score group in the combined GEO cohort. The correlation analysis further confirmed the close relationship between the risk score and the infiltration of most immune cell types (Fig. 7c, d).

Emerging studies support the crucial role of the TME in the response to immunotherapy [9, 29]. Moreover, studies have increasingly demonstrated that some immune checkpoint inhibitors, including the PD-1 inhibitors spartalizumab and pembrolizumab and the PD-L1 inhibitor nivolumab [4, 13–16], are effective in ATCs as salvage therapy. However, not all ATC patients could benefit from such treatment. Considering the positive correlation between the risk score and the expression of immune checkpoint molecules and the infiltration of immune cells, our study aimed to investigate the role of the risk score in the immunotherapy response of ATCs. Therefore, we first sought to obtain an immunotherapy database of ATC patients. However, after searching published immunotherapy datasets, no such cohort with complete prognosis data and expression profiles was obtained. As a complement, the IMvigor210 cohort and GSE63557 cohort, a dataset of urothelial cancer patients who received an anti-PD-L1 agent and a dataset of AB1-HA mesothelioma tumour mice treated with anti-CTLA4 therapy were used to investigate the value of the two-gene risk score in predicting the response to immunotherapy [20, 21]. We found that patients with a high risk score tended to have a better response to immunotherapy than those with a low risk score in the IMvigor210 cohort (p = 0.003; Fig. 7e). The number of mice responding to anti-CTLA4 agents was higher in the GSE63557 cohort, but the difference was not statistically significant (p = 0.18; Fig. 7g). Additionally, the risk score in patients with complete response (CR) to anti-PD-L1 therapy was higher than that in patients with progressive disease (PD) (p < 0.05) (Fig. 7f). Similarly, the risk score in mice that responded to anti-CTLA4 therapy was slightly higher than that in mice that did not respond (p < 0.05) (Fig. 7h).

### Molecular basis of the immune-related risk score signature

We further explored the differences between the high-risk score and low-risk score samples in the combined GEO cohort. Among the distinct signalling pathways enriched in the high-risk score group, the epithelial–mesenchymal transition, TNFα signalling, and some common immune-related signalling pathways, including the IL-6/JAK/STAT3 pathway, interferon alpha response, interferon gamma response and inflammatory response, obtained high NES values (Additional file 6: Figure S4).

## Discussion

Focusing on the mechanism of dedifferentiation promotes a better understanding of the high invasiveness of ATCs. Many previous studies have found that some molecules and pathways, such as STRN-ALK, P53, dendrogenin A, survivin and certain long noncoding RNAs, are involved in the dedifferentiation process of thyroid cells [30–33]. Indeed, publicly available resources, such as TCGA and GEO datasets, provide substantial clinical samples that enable more reliable and comprehensive studies. Thyroid carcinoma is the most common endocrine-related malignancy. Ma et al. [25] identified a metabolic gene signature indicative of the dedifferentiation of PTCs through transcriptome analyses. Similarly, Suh et al. [34] found that glucose metabolic profiles were correlated with differentiation in thyroid carcinoma. In addition, several researchers indicated that immune cells, especially macrophages, account for a large proportion of the tumour microenvironment in anaplastic thyroid carcinomas (ATCs) [12]. However, current studies investigating immune-related signatures in thyroid carcinoma by analysing public databases mainly focused on PTCs [26, 35, 36]. Therefore, we included ATC samples from the GEO database to identify an immune-related signature indicating the dedifferentiation of thyroid cells.

In this study, we initially focused on an investigation of IRGs in ATCs in comparison with those in normal tissues and PTCs. Through a joint analysis with TCGA cohort, two genes, namely, MMP9 and SDC2, were identified. MMP9 is a well-known oncogenic gene, and the enzyme encoded by this gene is involved in multiple processes in tumour progression, such as cancer proliferation, angiogenesis and metastasis [37–39]. Moreover, MMP9 has been reported to serve as an important inflammatory mediator in angiocardiopathy, infectious diseases and cancer [40–43]. The syndecan-2 protein, which is encoded by the SDC2 gene, is a type I transmembrane proteoglycan. The role of SDC2 depends on the type of tumour. It acts as an oncogene and contributes to disease progression in colon cancer, breast cancer and melanoma, while it serves as a metastasis suppressor in Lewis lung carcinoma [44–46]. Moreover, SDC2 has been found to be positively associated with the differentiation level and prognosis of neuroendocrine tumours, which is consistent with our findings [47].

By constructing a risk model based on univariate and multivariable Cox analyses, a significantly elevated risk score was observed in ATCs and low-differentiated PTCs. Notably, by performing a ROC analysis, we demonstrated that the risk score could distinguish the differentiation level in both the combined GEO cohort and TCGA cohort. By applying Kaplan–Meier curves, we found that the patients with high risk scores displayed a worse PFI than those with low risk scores. These findings suggest that the risk score could serve as an indicator of dedifferentiation and could contribute to the risk stratification and prediction of response to radioiodine therapy in thyroid carcinoma patients and act as a powerful biomarker for prognostic prediction.

Currently, immune checkpoint inhibitors, such as anti-PD-L1 and anti-CTLA-4 antibodies, elicit durable responses in some solid tumours, including ATCs [13–16]. In this study, we discovered a positive correlation between the risk score and the expression of important immune checkpoints or the enrichment of multiple immune cell types. Interestingly, by analysing two cohorts of patients with metastatic urothelial cancer who received anti-PD-L1 treatment (IMvigor210) and a mouse model treated with anti-CTLA-4 agent (GSE63557) [20, 21], we surprisingly observed that the samples with higher risk scores tended to have a better response to immune checkpoint therapy than the samples with low risk scores regardless of the weak statistical significance. This finding indicates that the risk score may not only contribute to the determination of differentiation and prognosis of thyroid cancer but also could play a role in predicting the infiltration of immune cells in the TME and the response to immunotherapy.

However, the current study has some limitations and drawbacks. First, due to the lack of survival information of the ATC patients in the GEO cohorts, the two risk-related genes were screened only in low-differentiated PTCs in TCGA cohort; thus, these results cannot completely reflect the results of ATCs. However, the risk scores and associations with TDS or immune checkpoints were verified in both cohorts. Second, the sample size in our study, especially of ATCs, was small; thus, the results obtained warrant further investigation. Third, the mechanism of the immune-related risk score in dedifferentiation is still undiscovered. In the future, we intend to carry out in vitro and in vivo experiments to verify our conclusions and further explore the mechanism of dedifferentiation mediated by the identified risk-related genes. To date, multiple antineoplastic drugs have been applied in ATCs [4], but reports of the efficacy of MMP9 inhibitors are lacking. Based on our findings, we speculate that MMP9 inhibitors alone or in combination with immunotherapy may be effective in the management of ATCs, but this hypothesis requires further verification.

## Conclusions

Our findings indicate the potential value of an immune-related risk score composed of MMP9 and SDC2 for predicting dedifferentiation and survival in thyroid carcinoma. The risk score may also help predict the infiltration of immune cells and the response to immunotherapy. Thus, the risk score could be used as a powerful biomarker of differentiation and prognosis in patients with thyroid carcinoma. Additionally, the included risk-related genes could be further explored as new therapeutic targets for the treatment of ATCs.

## Supplementary Information

**Additional file 1: Figure S1.** Functional differences in IRGs between ATCs and PTCs or normal tissues. GSEA of IRGs showed the activated pathways, except for the inflammatory response, in ATCs compared with those in normal tissues (**a**) and PTCs (**b**).

**Additional file 2: Figure S2.** GSEA identified differentiation-associated immune signalling pathways.

**Additional file 3: Table S1.** Univariate analysis of prognosis-associated risk factors for low-differentiated PTCs in TCGA database.

**Additional file 4: Figure S3.** The expression of TG, PLAUR and FGFR2 was detected by using immunohistochemistry.

**Additional file 5: Table S2.** The details of the IHC score for each protein.

**Additional file 6: Figure S4.** Molecular basis of the immune-related risk score signature. (**a**) Volcano plot of the GSEA of the low-risk-score versus high-risk-score samples in the combined GEO cohort. (**b**) GSEA of the biological signalling pathways significantly enriched in the high-risk-score samples in the combined GEO cohort.

## Data Availability

The datasets analysed during the current study are available in the GEO database, TCGA database and the Immport website at [https://www.ncbi.nlm.nih.gov/geo/, https://xenabrowser.net/datapages/, https://immport.org/shared/home].

## Abbreviations

ATC: Anaplastic thyroid carcinoma
AUC: Area under curve
CTLA4: Cytotoxic T-lymphocyte protein 4
GEO: Gene Expression Omnibus
GSEA: Gene set enrichment analysis
IRG: Immune-related gene
IDO1: Indoleamine 2,3-dioxygenase 1
IHC: Immunohistochemistry
MMP9: Matrix metalloproteinase-9
NES: Normalized enrichment score
PTC: Papillary thyroid carcinoma
PFI: Progression-free interval
PCA: Principal component analysis
PD-L1: Programmed cell death 1 ligand 1 (also called CD274)
ROC: Receiver operating characteristic
ssGSEA: Single sample gene set enrichment analysis
SDC2: Syndecan-2
TDS: Thyroid differentiation score
TME: Tumor microenvironment
TCGA: The Cancer Genome Atlas
TIM-3: T cell immunoglobulin-3 (also called HAVCR2, hepatitis a virus cellular receptor 2)
