# Supplementary figures and images for: Identification of an immune-related signature indicating the dedifferentiation of thyroid cells
Source: Cancer Cell Int. 2021 Apr 23;21:231. doi: 10.1186/s12935-021-01939-3 (PMC8067302; doi:10.1186/s12935-021-01939-3)

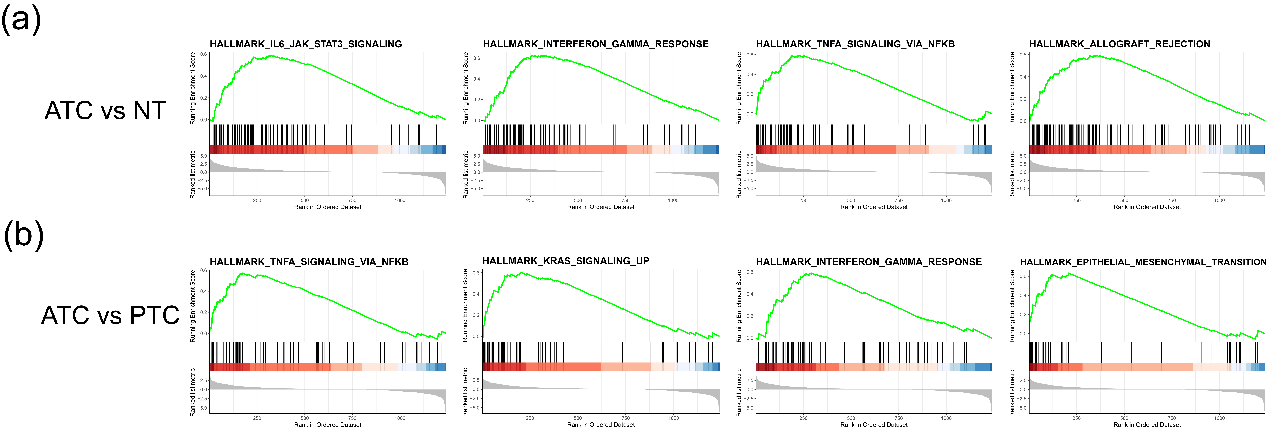

Supplement: Supplementary file 1 — Additional file 1: Figure S1. Functional differences in IRGs between ATCs and PTCs or normal tissues. GSEA of IRGs showed the activated pathways, except for the inflammatory response, in ATCs compared with those in normal tissues (a) and PTCs (b). [file 12935_2021_1939_MOESM1_ESM.docx]

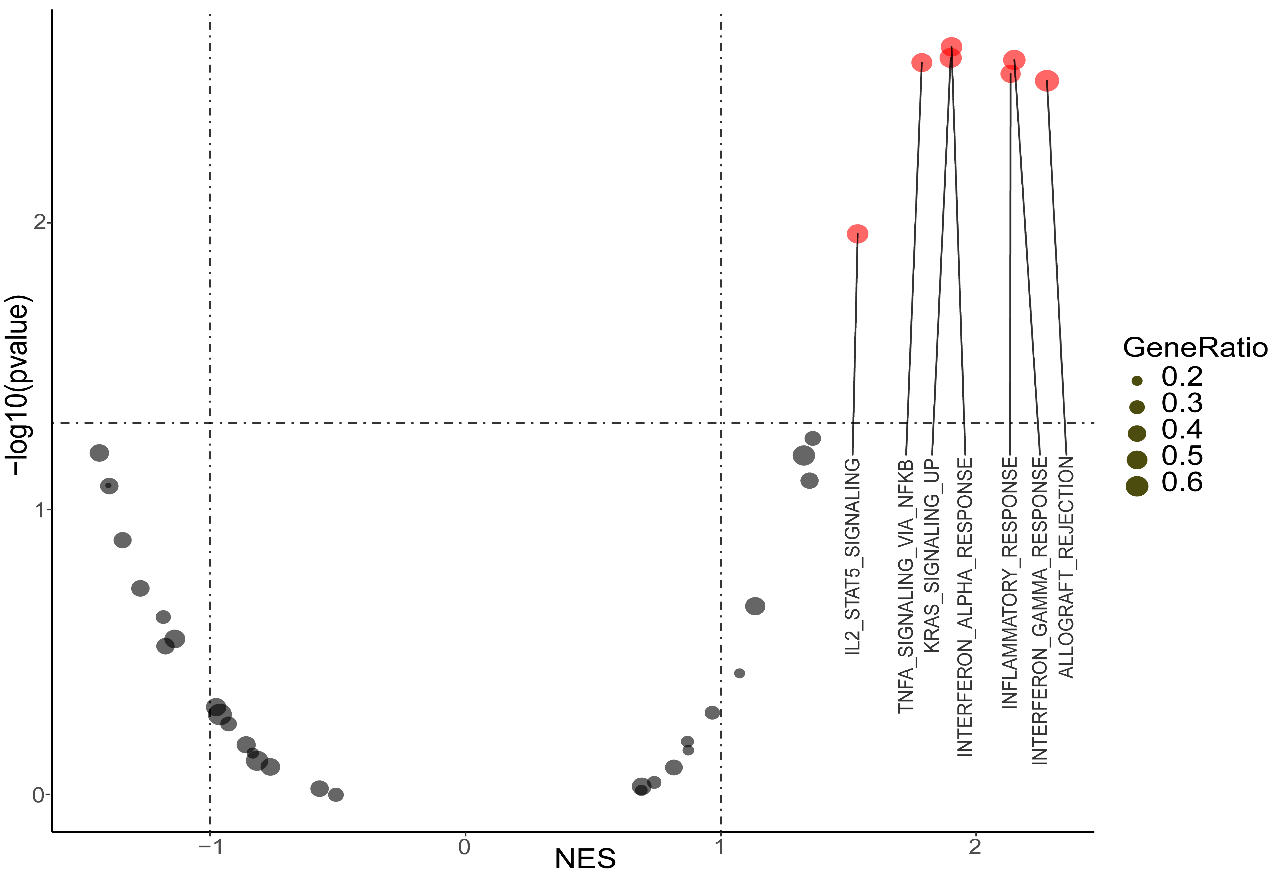

Supplement: Supplementary file 2 — Additional file 2: Figure S2. GSEA identified differentiation-associated immune signalling pathways. [file 12935_2021_1939_MOESM2_ESM.docx]

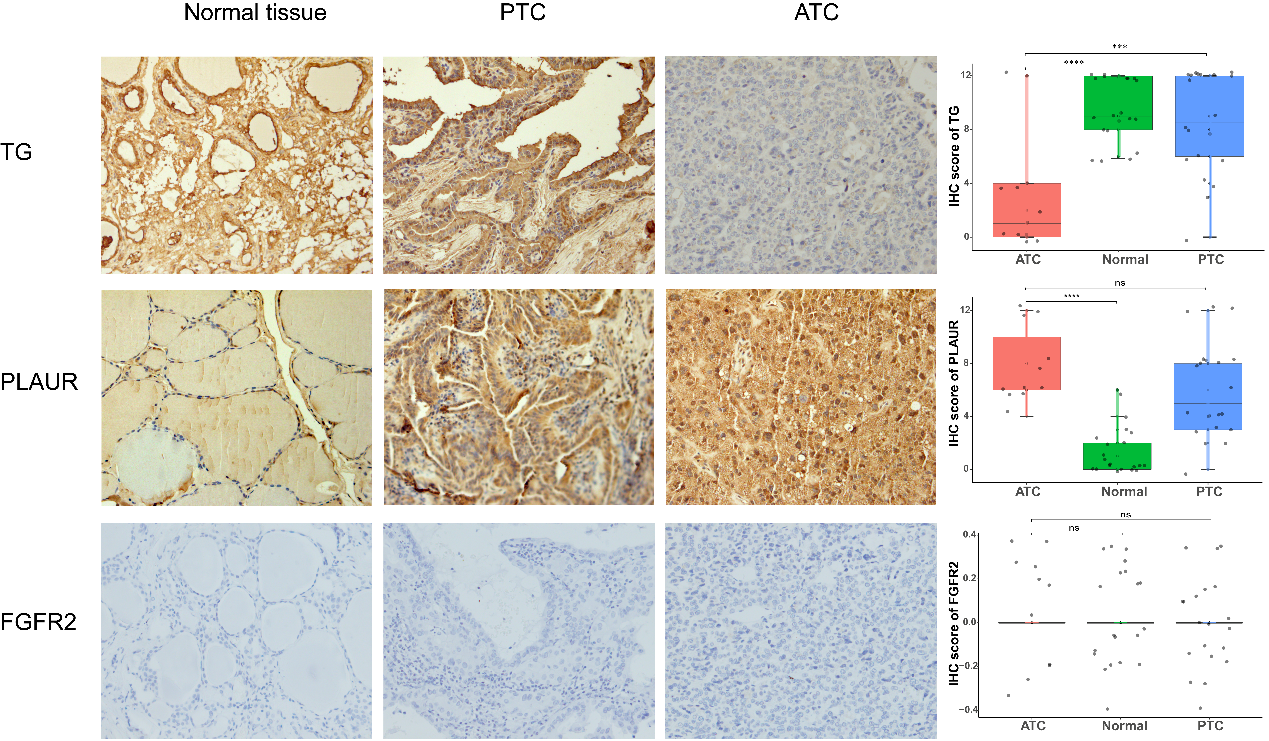

Supplement: Supplementary file 4 — Additional file 4: Figure S3. The expression of TG, PLAUR and FGFR2 was detected by using immunohistochemistry. [file 12935_2021_1939_MOESM4_ESM.docx]

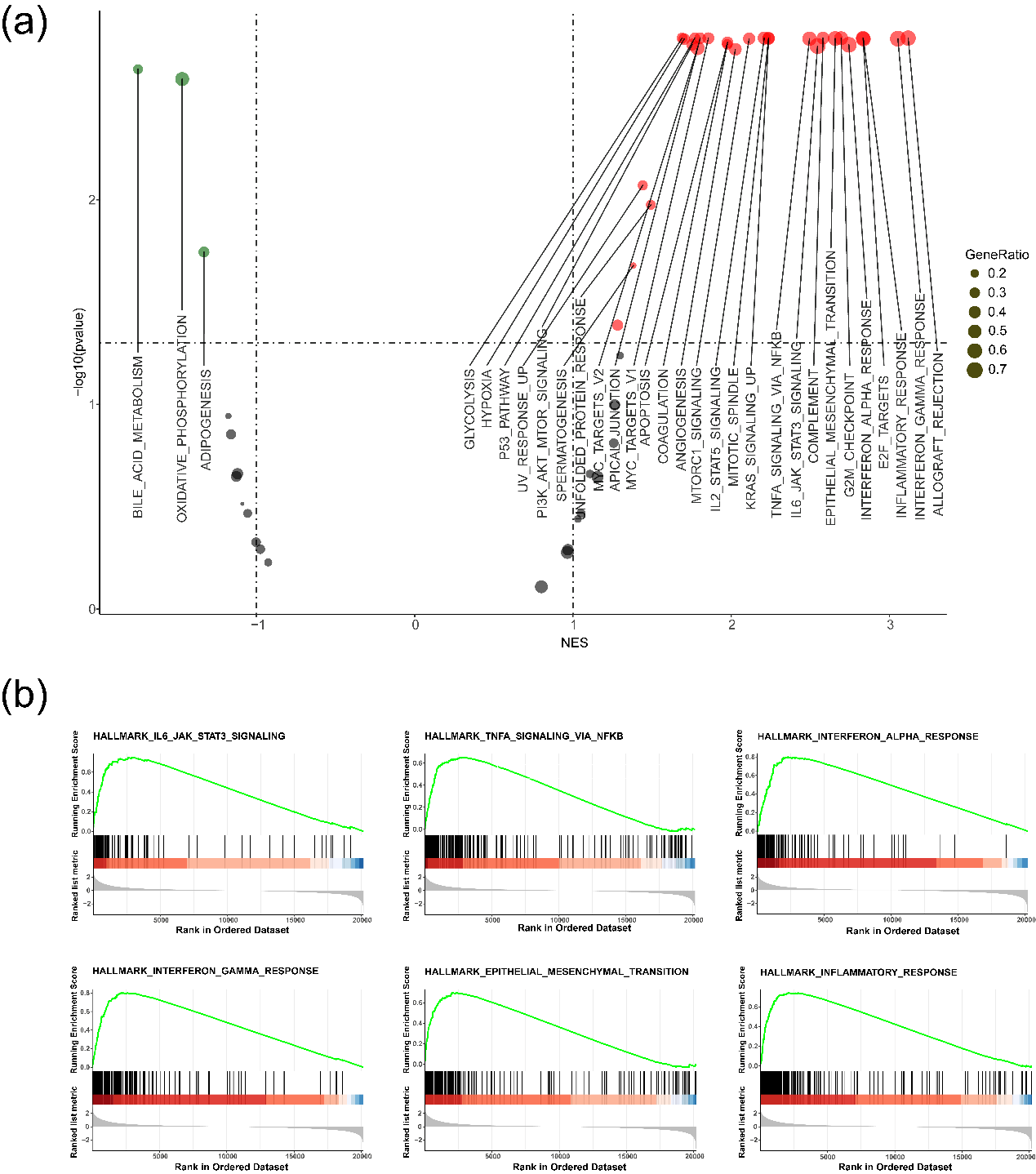

Supplement: Supplementary file 6 — Additional file 6: Figure S4. Molecular basis of the immune-related risk score signature. (a) Volcano plot of the GSEA of the low-risk-score versus high-risk-score samples in the combined GEO cohort. (b) GSEA of the biological signalling pathways significantly enriched in the high-risk-score samples in the combined GEO cohort. [file 12935_2021_1939_MOESM6_ESM.docx]
