# Supplementary material for: Identification of an immune-related signature indicating the dedifferentiation of thyroid cells
Source: Cancer Cell Int. 2021 Apr 23;21:231. doi: 10.1186/s12935-021-01939-3 (PMC8067302; doi:10.1186/s12935-021-01939-3)
Supplement: Supplementary file 3 — Additional file 3: Table S1. Univariate analysis of prognosis-associated risk factors for low-differentiated PTCs in TCGA database. [file 12935_2021_1939_MOESM3_ESM.docx]

**Additional file 3: Table S1** Univariate analysis of prognosis-associated risk factors for low-differentiated PTCs in TCGA database.

|  | Univariate analysis | |
| --- | --- | --- |
| Variable | HR 95%CI | p |
| Age |  |  |
| ＜60 | 1 |  |
| ≥60 | 1.015 0.994-1.036 | 0.167 |
| Gender |  |  |
| Male | 1 |  |
| Female | 0.992 0.464-2.117 | 0.983 |
| **Stage** |  |  |
| Ⅰ/Ⅱ | 1 |  |
| Ⅲ/Ⅳ | 2.212 1.135-4.311 | **0.020** |
| **Local invasion** |  |  |
| T1/T2 | 1 |  |
| T3/T4 | 2.367 1.177-4.758 | **0.016** |
| Lymph node metastasis |  |  |
| N0 | 1 |  |
| N1 | 1.826 0.820-4.076 | 0.140 |
| **Distant metastasis** |  |  |
| M0 | 1 |  |
| M1 | 6.485 1.853-22.698 | **0.003** |
| Extrathyroid extension(ETE) |  |  |
| No | 1 |  |
| Yes | 1.633 0.833-3.204 | 0.153 |
| IRGs |  |  |
| ANGPTL2 | 1.122 0.766-1.644 | 0.555 |
| ANGPTL4 | 0.802 0.597-1.077 | 0.142 |
| BIRC5 | 1.269 0.921-1.747 | 0.145 |
| BMPR1B | 1.100 0.890-1.358 | 0.377 |
| C5AR1 | 1.131 0.838-1.527 | 0.420 |
| CCR1 | 0.924 0.650-1.313 | 0.659 |
| CD209 | 1.004 0.773-1.305 | 0.973 |
| CSF1R | 1.017 0.708-1.462 | 0.926 |
| CXCL5 | 1.078 0.909-1.279 | 0.388 |
| CXCL6 | 1.032 0.747-1.428 | 0.847 |
| DKK1 | 0.895 0.711-1.128 | 0.347 |
| FPR1 | 1.024 0.781-1.341 | 0.865 |
| GREM1 | 1.066 0.933-1.218 | 0.349 |
| HGF | 0.990 0.775-1.264 | 0.935 |
| IL2RA | 1.120 0.886-1.417 | 0.344 |
| LCP2 | 0.946 0.664-1.348 | 0.760 |
| LTBP1 | 1.179 0.938-1.482 | 0.158 |
| MICB | 0.977 0.701-1.361 | 0.890 |
| MMP12 | 1.090 0.930-1.277 | 0.287 |
| **MMP9** | 1.393 1.119-1.735 | **0.003** |
| MSR1 | 1.062 0.788-1.429 | 0.694 |
| NRAS | 1.673 0.771-3.634 | 0.193 |
| OSMR | 1.120 0.841-1.492 | 0.437 |
| OXTR | 1.063 0.729-1.551 | 0.750 |
| PDGFRA | 1.025 0.862-1.220 | 0.778 |
| PI15 | 1.175 0.984-1.404 | 0.075 |
| PIK3R5 | 1.013 0.744-1.380 | 0.933 |
| **PLAUR** | 1.339 1.006-1.782 | **0.045** |
| PLTP | 0.936 0.681-1.288 | 0.686 |
| PROCR | 0.994 0.618-1.601 | 0.981 |
| PTGER2 | 0.928 0.720-1.196 | 0.565 |
| PTGFR | 1.118 0.908-1.378 | 0.294 |
| PTX3 | 1.250 0.869-1.798 | 0.228 |
| SPP1 | 1.139 0.921-1.408 | 0.231 |
| THBS1 | 1.117 0.900-1.386 | 0.316 |
| TLR4 | 0.869 0.555-1.358 | 0.537 |
| AKT2 | 1.213 0.516-2.850 | 0.658 |
| ANGPTL1 | 0.852 0.676-1.074 | 0.175 |
| ARG2 | 1.037 0.737-1.459 | 0.834 |
| BMP7 | 0.899 0.470-1.718 | 0.747 |
| BTC | 1.353 0.947-1.935 | 0.097 |
| CAT | 0.731 0.439-1.215 | 0.227 |
| CDH1 | 0.986 0.514-1.893 | 0.967 |
| CLDN4 | 1.136 0.618-2.088 | 0.681 |
| CMTM4 | 1.268 0.705-2.280 | 0.428 |
| CYLD | 1.069 0.491-2.327 | 0.866 |
| DUOX1 | 1.027 0.683-1.545 | 0.897 |
| DUOX2 | 0.893 0.723-1.103 | 0.293 |
| ESR1 | 0.934 0.774-1.128 | 0.479 |
| **FGFR2** | 0.668 0.454-0.984 | **0.041** |
| FOS | 0.938 0.766-1.149 | 0.534 |
| IL6ST | 1.087 0.911-1.296 | 0.353 |
| NR3C2 | 1.215 0.688-2.144 | 0.503 |
| PAK1 | 0.902 0.418-1.946 | 0.793 |
| PDGFRL | 1.058 0.544-2.059 | 0.868 |
| PIK3CB | 0.791 0.385-1.627 | 0.524 |
| PIK3R1 | 1.127 0.634-2.002 | 0.684 |
| PRKCQ | 0.603 0.346-1.050 | 0.074 |
| PSMC2 | 1.817 0.406-8.140 | 0.435 |
| RORA | 1.154 0.679-1.959 | 0.596 |
| RORC | 0.703 0.419-1.180 | 0.182 |
| S100A14 | 1.360 0.880-2.102 | 0.166 |
| **SDC2** | 0.575 0.386-0.856 | **0.006** |
| SEMA6D | 0.995 0.742-1.334 | 0.972 |
| SORT1 | 1.004 0.396-2.545 | 0.993 |
| TEK | 0.845 0.607-1.175 | 0.317 |
| **TG** | 0.823 0.686-0.987 | **0.035** |
| TSHR | 0.981 0.718-1.341 | 0.906 |

HR, hazard ratio; CI, confidence interval;

IRGs were evaluated as continuous variables in univariate Cox regression analysis;

Variables with statistical p < 0.05 are in bold.
