# Supplementary material for: Identification of an immune-related signature indicating the dedifferentiation of thyroid cells
Source: Cancer Cell Int. 2021 Apr 23;21:231. doi: 10.1186/s12935-021-01939-3 (PMC8067302; doi:10.1186/s12935-021-01939-3)
Supplement: Supplementary file 5 — Additional file 5: Table S2. The details of the IHC score for each protein. [file 12935_2021_1939_MOESM5_ESM.docx]

**Additional file 5: Table S2** The details of the IHC score for each protein.

**Statistical table of MMP9 protein expression**

| histopathological type | number | expression intensity  (0=no;  1=weak; 2=moderate; 3=strong) | percentage  (0=0~5%; 1=6~25%; 2=26~50%; 3=51~75%; 4=75~100%) | IHC score  (expression intensity  * percentage) |
| --- | --- | --- | --- | --- |
| Normal tissue | 1 | 0 | 0 | 0 |
|  | 2 | 0 | 0 | 0 |
|  | 3 | 0 | 0 | 0 |
|  | 4 | 0 | 0 | 0 |
|  | 5 | 0 | 0 | 0 |
|  | 6 | 1 | 0 | 0 |
|  | 7 | 0 | 0 | 0 |
|  | 8 | 0 | 0 | 0 |
|  | 9 | 2 | 2 | 4 |
|  | 10 | 0 | 0 | 0 |
|  | 11 | 2 | 0 | 0 |
|  | 12 | 3 | 0 | 0 |
|  | 13 | 0 | 0 | 0 |
|  | 14 | 0 | 0 | 0 |
|  | 15 | 0 | 0 | 0 |
|  | 16 | 0 | 0 | 0 |
|  | 17 | 0 | 0 | 0 |
|  | 18 | 0 | 0 | 0 |
|  | 19 | 0 | 0 | 0 |
|  | 20 | 3 | 0 | 0 |
| PTC | 1 | 2 | 0 | 0 |
|  | 2 | 0 | 0 | 0 |
|  | 3 | 0 | 0 | 0 |
|  | 4 | 0 | 0 | 0 |
|  | 5 | 0 | 0 | 0 |
|  | 6 | 0 | 0 | 0 |
|  | 7 | 0 | 0 | 0 |
|  | 8 | 2 | 0 | 0 |
|  | 9 | 0 | 0 | 0 |
|  | 10 | 0 | 0 | 0 |
|  | 11 | 0 | 0 | 0 |
|  | 12 | 3 | 0 | 0 |
|  | 13 | 2 | 0 | 0 |
|  | 14 | 0 | 0 | 0 |
|  | 15 | 2 | 0 | 0 |
|  | 16 | 2 | 1 | 2 |
|  | 17 | 2 | 0 | 0 |
|  | 18 | 0 | 0 | 0 |
|  | 19 | 3 | 1 | 3 |
|  | 20 | 2 | 0 | 0 |
| ATC | 1 | 3 | 0 | 0 |
|  | 2 | 3 | 2 | 6 |
|  | 3 | 3 | 2 | 6 |
|  | 4 | 2 | 1 | 2 |
|  | 5 | 3 | 3 | 9 |
|  | 6 | 2 | 1 | 2 |
|  | 7 | 3 | 1 | 3 |
|  | 8 | 3 | 2 | 6 |
|  | 9 | 3 | 0 | 0 |
|  | 10 | 3 | 2 | 6 |
|  | 11 | 3 | 2 | 6 |

**Statistical table of SDC2 protein expression**

| histopathological type | number | expression intensity  (0=no;  1=weak; 2=moderate; 3=strong) | percentage  (0=0~5%; 1=6~25%; 2=26~50%; 3=51~75%; 4=75~100%) | IHC score  (expression intensity  * percentage) |
| --- | --- | --- | --- | --- |
| Normal tissue | 1 | 3 | 1 | 3 |
|  | 2 | 2 | 3 | 6 |
|  | 3 | 3 | 2 | 6 |
|  | 4 | 3 | 4 | 12 |
|  | 5 | 3 | 4 | 12 |
|  | 6 | 3 | 4 | 12 |
|  | 7 | 3 | 4 | 12 |
|  | 8 | 3 | 4 | 12 |
|  | 9 | 3 | 4 | 12 |
|  | 10 | 3 | 4 | 12 |
|  | 11 | 3 | 4 | 12 |
|  | 12 | 3 | 4 | 12 |
|  | 13 | 3 | 4 | 12 |
|  | 14 | 3 | 4 | 12 |
|  | 15 | 3 | 4 | 12 |
|  | 16 | 3 | 4 | 12 |
|  | 17 | 3 | 4 | 12 |
|  | 18 | 3 | 3 | 9 |
|  | 19 | 3 | 2 | 6 |
|  | 20 | 3 | 4 | 12 |
| PTC | 1 | 1 | 4 | 4 |
|  | 2 | 1 | 3 | 3 |
|  | 3 | 0 | 0 | 0 |
|  | 4 | 3 | 4 | 12 |
|  | 5 | 3 | 4 | 12 |
|  | 6 | 3 | 4 | 12 |
|  | 7 | 2 | 4 | 8 |
|  | 8 | 3 | 4 | 12 |
|  | 9 | 3 | 4 | 12 |
|  | 10 | 3 | 4 | 12 |
|  | 11 | 3 | 4 | 12 |
|  | 12 | 3 | 4 | 12 |
|  | 13 | 3 | 4 | 12 |
|  | 14 | 3 | 4 | 12 |
|  | 15 | 3 | 4 | 12 |
|  | 16 | 0 | 0 | 0 |
|  | 17 | 0 | 0 | 0 |
|  | 18 | 3 | 4 | 12 |
|  | 19 | 2 | 4 | 8 |
|  | 20 | 3 | 4 | 12 |
| ATC | 1 | 1 | 2 | 2 |
|  | 2 | 0 | 0 | 0 |
|  | 3 | 0 | 0 | 0 |
|  | 4 | 0 | 0 | 0 |
|  | 5 | 1 | 2 | 2 |
|  | 6 | 1 | 1 | 1 |
|  | 7 | 2 | 1 | 2 |
|  | 8 | 1 | 4 | 4 |
|  | 9 | 2 | 4 | 8 |
|  | 10 | 3 | 4 | 12 |
|  | 11 | 2 | 4 | 8 |

**Statistical table of TG protein expression**

| histopathological type | number | expression intensity  (0=no;  1=weak; 2=moderate; 3=strong) | percentage  (0=0~5%; 1=6~25%; 2=26~50%; 3=51~75%; 4=75~100%) | IHC score  (expression intensity  * percentage) |
| --- | --- | --- | --- | --- |
| Normal tissue | 1 | 3 | 2 | 6 |
|  | 2 | 3 | 3 | 9 |
|  | 3 | 3 | 3 | 9 |
|  | 4 | 3 | 2 | 6 |
|  | 5 | 3 | 3 | 9 |
|  | 6 | 3 | 4 | 12 |
|  | 7 | 3 | 3 | 9 |
|  | 8 | 3 | 4 | 12 |
|  | 9 | 2 | 4 | 8 |
|  | 10 | 2 | 3 | 6 |
|  | 11 | 3 | 4 | 12 |
|  | 12 | 3 | 4 | 12 |
|  | 13 | 3 | 4 | 12 |
|  | 14 | 3 | 4 | 12 |
|  | 15 | 3 | 3 | 9 |
|  | 16 | 3 | 4 | 12 |
|  | 17 | 2 | 4 | 8 |
|  | 18 | 3 | 2 | 6 |
|  | 19 | 3 | 4 | 12 |
|  | 20 | 3 | 3 | 9 |
| PTC | 1 | 1 | 4 | 4 |
|  | 2 | 2 | 4 | 8 |
|  | 3 | 1 | 4 | 4 |
|  | 4 | 1 | 3 | 3 |
|  | 5 | 3 | 4 | 12 |
|  | 6 | 2 | 3 | 6 |
|  | 7 | 3 | 4 | 12 |
|  | 8 | 2 | 3 | 6 |
|  | 9 | 3 | 4 | 12 |
|  | 10 | 3 | 4 | 12 |
|  | 11 | 0 | 0 | 0 |
|  | 12 | 3 | 4 | 12 |
|  | 13 | 3 | 4 | 12 |
|  | 14 | 3 | 4 | 12 |
|  | 15 | 2 | 4 | 8 |
|  | 16 | 2 | 3 | 6 |
|  | 17 | 3 | 4 | 12 |
|  | 18 | 3 | 3 | 9 |
|  | 19 | 3 | 4 | 12 |
|  | 20 | 2 | 4 | 8 |
| ATC | 1 | 0 | 0 | 0 |
|  | 2 | 0 | 0 | 0 |
|  | 3 | 1 | 1 | 1 |
|  | 4 | 0 | 0 | 0 |
|  | 5 | 2 | 0 | 0 |
|  | 6 | 1 | 4 | 4 |
|  | 7 | 1 | 2 | 2 |
|  | 8 | 1 | 4 | 4 |
|  | 9 | 1 | 4 | 4 |
|  | 10 | 3 | 4 | 12 |
|  | 11 | 0 | 0 | 0 |

**Statistical table of PLAUR protein expression**

| histopathological type | number | expression intensity  (0=no;  1=weak; 2=moderate; 3=strong) | percentage  (0=0~5%; 1=6~25%; 2=26~50%; 3=51~75%; 4=75~100%) | IHC score  (expression intensity  * percentage) |
| --- | --- | --- | --- | --- |
| Normal tissue | 1 | 0 | 0 | 0 |
|  | 2 | 1 | 0 | 0 |
|  | 3 | 1 | 2 | 2 |
|  | 4 | 1 | 3 | 3 |
|  | 5 | 1 | 1 | 1 |
|  | 6 | 1 | 3 | 3 |
|  | 7 | 3 | 0 | 0 |
|  | 8 | 0 | 0 | 0 |
|  | 9 | 2 | 3 | 6 |
|  | 10 | 2 | 0 | 0 |
|  | 11 | 2 | 1 | 2 |
|  | 12 | 1 | 1 | 1 |
|  | 13 | 1 | 0 | 0 |
|  | 14 | 1 | 0 | 0 |
|  | 15 | 0 | 0 | 0 |
|  | 16 | 2 | 1 | 2 |
|  | 17 | 2 | 2 | 4 |
|  | 18 | 1 | 0 | 0 |
|  | 19 | 1 | 0 | 0 |
|  | 20 | 1 | 0 | 0 |
| PTC | 1 | 1 | 3 | 3 |
|  | 2 | 1 | 4 | 4 |
|  | 3 | 2 | 4 | 8 |
|  | 4 | 1 | 4 | 4 |
|  | 5 | 1 | 4 | 4 |
|  | 6 | 1 | 3 | 3 |
|  | 7 | 0 | 0 | 0 |
|  | 8 | 3 | 4 | 12 |
|  | 9 | 2 | 4 | 8 |
|  | 10 | 2 | 4 | 8 |
|  | 11 | 1 | 3 | 3 |
|  | 12 | 2 | 3 | 6 |
|  | 13 | 2 | 4 | 8 |
|  | 14 | 2 | 4 | 8 |
|  | 15 | 3 | 4 | 12 |
|  | 16 | 3 | 4 | 12 |
|  | 17 | 1 | 2 | 2 |
|  | 18 | 1 | 2 | 2 |
|  | 19 | 2 | 4 | 8 |
|  | 20 | 1 | 4 | 4 |
| ATC | 1 | 3 | 2 | 6 |
|  | 2 | 3 | 4 | 12 |
|  | 3 | 2 | 4 | 8 |
|  | 4 | 3 | 4 | 12 |
|  | 5 | 2 | 3 | 6 |
|  | 6 | 3 | 4 | 12 |
|  | 7 | 3 | 2 | 6 |
|  | 8 | 2 | 4 | 8 |
|  | 9 | 2 | 3 | 6 |
|  | 10 | 2 | 3 | 6 |
|  | 11 | 1 | 4 | 4 |

**Statistical table of FGFR2 protein expression**

| histopathological type | number | expression intensity  (0=no;  1=weak; 2=moderate; 3=strong) | percentage  (0=0~5%; 1=6~25%; 2=26~50%; 3=51~75%; 4=75~100%) | IHC score  (expression intensity  * percentage) |
| --- | --- | --- | --- | --- |
| Normal tissue | 1 | 1 | 0 | 0 |
|  | 2 | 0 | 0 | 0 |
|  | 3 | 1 | 0 | 0 |
|  | 4 | 0 | 0 | 0 |
|  | 5 | 1 | 0 | 0 |
|  | 6 | 2 | 0 | 0 |
|  | 7 | 0 | 0 | 0 |
|  | 8 | 0 | 0 | 0 |
|  | 9 | 0 | 0 | 0 |
|  | 10 | 0 | 0 | 0 |
|  | 11 | 0 | 0 | 0 |
|  | 12 | 1 | 0 | 0 |
|  | 13 | 0 | 0 | 0 |
|  | 14 | 0 | 0 | 0 |
|  | 15 | 0 | 0 | 0 |
|  | 16 | 0 | 0 | 0 |
|  | 17 | 0 | 0 | 0 |
|  | 18 | 0 | 0 | 0 |
|  | 19 | 0 | 0 | 0 |
|  | 20 | 0 | 0 | 0 |
| PTC | 1 | 0 | 0 | 0 |
|  | 2 | 0 | 0 | 0 |
|  | 3 | 0 | 0 | 0 |
|  | 4 | 0 | 0 | 0 |
|  | 5 | 1 | 0 | 0 |
|  | 6 | 0 | 0 | 0 |
|  | 7 | 0 | 0 | 0 |
|  | 8 | 0 | 0 | 0 |
|  | 9 | 0 | 0 | 0 |
|  | 10 | 0 | 0 | 0 |
|  | 11 | 0 | 0 | 0 |
|  | 12 | 0 | 0 | 0 |
|  | 13 | 0 | 0 | 0 |
|  | 14 | 1 | 0 | 0 |
|  | 15 | 0 | 0 | 0 |
|  | 16 | 0 | 0 | 0 |
|  | 17 | 0 | 0 | 0 |
|  | 18 | 0 | 0 | 0 |
|  | 19 | 0 | 0 | 0 |
|  | 20 | 0 | 0 | 0 |
| ATC | 1 | 0 | 0 | 0 |
|  | 2 | 0 | 0 | 0 |
|  | 3 | 0 | 0 | 0 |
|  | 4 | 0 | 0 | 0 |
|  | 5 | 1 | 0 | 0 |
|  | 6 | 0 | 0 | 0 |
|  | 7 | 0 | 0 | 0 |
|  | 8 | 0 | 0 | 0 |
|  | 9 | 0 | 0 | 0 |
|  | 10 | 0 | 0 | 0 |
|  | 11 | 0 | 0 | 0 |
